# Building a Risk Scoring Model for ARDS in Lung Adenocarcinoma Patients Using Machine Learning Algorithms

**DOI:** 10.1111/jcmm.70280

**Published:** 2024-12-10

**Authors:** Erchun Hong, Yunyun Sun, Yongming Qin, Wenjun Zhao, Yanzi Qin, Xincan Li, Liang Zhang

**Affiliations:** ^1^ Department of Emergency Medicine, Bengbu Third People's Hospital Bengbu Medical University Bengbu Anhui Province China; ^2^ Department of Neurology, Bengbu Third People's Hospital Bengbu Medical University Bengbu Anhui Province China; ^3^ Department of Pathology Bengbu Medical University Bengbu Anhui Province China

**Keywords:** acute respiratory distress syndrome, biomarkers, lung adenocarcinoma, risk scoring model

## Abstract

Lung adenocarcinoma (LUAD), the predominant form of non‐small‐cell lung cancer, is frequently complicated by acute respiratory distress syndrome (ARDS), which increases mortality risks. Investigating the prognostic implications of ARDS‐related genes in LUAD is crucial for improving clinical outcomes. Data from TCGA, GEO and GTEx were used to identify 276 ARDS‐related genes in LUAD via differential expression analysis. Univariate Cox regression, consensus clustering and machine learning algorithms were used to develop a prognostic risk scoring model. Functional enrichment, immune infiltration analyses, copy number variations and mutational burdens were examined, and the results were validated at the single‐cell level. ARDS‐related genes significantly impact the prognosis of LUAD patients. A machine learning‐based risk scoring model accurately predicted survival rates. Functional enrichment and immune infiltration analyses revealed that these genes are primarily involved in cell cycle regulation and immune cell infiltration. Single‐cell expression data supported these findings, and the assessments of copy number variations and mutational burdens highlighted distinct genetic characteristics. This study establishes the prognostic relevance of ARDS‐associated genes in LUAD and provides potential biomarkers for personalized therapy and prognosis. Future studies will validate these findings and explore their clinical applications.

## 1 Introduction

Lung adenocarcinoma (LUAD), the predominant form of non‐small‐cell lung cancer (NSCLC), is a leading cause of cancer incidence and mortality globally, posing a significant threat to public health [1]. LUAD originates from the glandular tissue of the lungs, characterized by tumour cells growing along the alveolar walls, forming papillary or glandular structures [2]. Due to the lack of obvious early symptoms, many patients are already in advanced stages when diagnosed, posing significant challenges to the treatment and prognosis of LUAD [1, 3, 4]. According to global cancer statistics, every year there are millions of new cases diagnosed with LUAD, resulting in poor treatment outcomes and a grim prognosis [4, 5].

The diagnosis of LUAD mainly relies on imaging studies, histopathological evaluation and molecular biomarker detection. With the advancement of precision medicine, personalized treatment strategies have gradually become a research focus, in which genomic and transcriptomic analyses provide important information for molecular subtyping and the discovery of therapeutic targets for LUAD [6, 7]. However, the treatment response and prognosis of LUAD are influenced by multiple factors, including the biological characteristics of the tumour, the patient's immune status and the tumour microenvironment [8].

Acute respiratory distress syndrome (ARDS) is not only common in patients with LUAD but may also occur as a complication during the treatment process of LUAD, such as lung injury caused by chemotherapy or radiotherapy [9]. In addition, the occurrence of ARDS can significantly increase the risk of death for patients [10]. In addition, the occurrence of ARDS is closely related to the prognosis of patients with LUAD, and it can significantly increase the risk of death for patients [11]. For example, radiotherapy may lead to radiation pneumonitis, a severe pulmonary complication similar to ARDS. Lung cancer patients may have an immune‐suppressed state, which not only affects the growth and spread of tumours but may also impact the lung's ability to defend against infections, thereby increasing the risk of ARDS. In the context of LUAD, molecular biomarkers of ARDS may be related to the invasiveness of the tumour, immune evasion and responsiveness to treatment.

The possible mechanisms of ARDS, and how it influences LUAD could be developed from its role in inflammation and immune regulation. ARDS has been associated with an intense inflammatory response whose sequel may then create a microenvironment favourable for the development and dissemination of LUAD. The inflammatory milieu, in particular, can support processes such as angiogenesis and epithelial–mesenchymal transition (EMT), which provides waves through immune suppression, all favourable progresses for tumour progression and metastasis. Understanding whether ARDS‐related genes play a role in the outcome of LUAD would provide new biomarkers and treatment targets.

## 2 Methods

### 2.1 Data Source

The bulk RNA expression data and clinical profiles for LUAD utilized in this analysis were derived from The Cancer Genome Atlas (TCGA) [12] and the Gene Expression Omnibus (GEO) database. In detail, the dataset encompassed 516 patient cases from TCGA, an additional 117 cases from the GSE13213 study, 452 cases from the GSE31210 study and 347 normal tissue samples from the Genotype‐Tissue Expression (GTEx) project. Furthermore, single‐cell RNA sequencing (scRNA‐seq) data from LUAD tissues were extracted from the GSE131907 dataset (208,506 cells derived from 58 LUADs from 44 patients).

### 2.2 ARDS‐Related Lung Adenocarcinoma Differential Genes

A total of 6063 ARDS‐related genes were obtained from GeneCard (https://www.genecards.org/), MalaCards (https://www.malacards.org/) and Harmonizome (https://maayanlab.cloud/Harmonizome/). By merging bulk analysis data of LUAD with normal tissue samples for differential analysis, a total of 276 ARDS‐related differential genes were identified (logFC filter = 1, FDR filter = 0.05). By setting a logFC threshold of 1, the analysis focuses on genes that show at least a twofold change in expression, which is often considered a biologically relevant threshold. By using an FDR correction, the analysis accounts for the multiple testing problems inherent in genomic studies, where thousands of genes are tested simultaneously. Further analysis involved single‐factor COX analysis and expression correlation analysis with clinical information.

### 2.3 Consensus Clustering Analysis

A consensus clustering analysis was conducted on ARDS‐related genes that were significantly associated with patient prognosis. Utilizing the ‘ConsensusClusterPlus’ R package in conjunction with the *k*‐means clustering algorithm, an unsupervised approach was taken to cluster patients into distinct groups based on their gene expression profiles. This process involved 1000 selecting 80% of the dataset for analysis, evaluating the item‐consensus plot, applying the PAC score and assessing the curve. Ultimately, two distinct clusters were identified: the ‘ARDS‐related’ and ‘non‐ARDS‐related’ groups. Subsequently, Kaplan–Meier survival analysis was applied to these two groups to compare their overall survival rates.

### 2.4 Machine Learning, Model Building and Validation

For the analysis of LUAD, datasets were bifurcated into TCGA and GEO cohorts to develop and train machine learning models, using a suite of 10 distinct algorithms and 101 algorithmic combinations [13, 14]. The methodologies encompassed random survival forests (RSF), elastic net (Enet), lasso, ridge regression, stepwise Cox, CoxBoost, partial least squares regression for Cox models (plsRcox), super principal components (SuperPC), gradient boosting machines (GBM) and survival support vector machines (survival‐SVM). Lasso is a regularization method that performs feature selection by shrinking some coefficients to zero, which is beneficial in high‐dimensional data settings where the number of features may exceed the number of observations. The model selection criterion was based on the highest average Harrell's concordance index (C‐index) across both TCGA and GEO datasets. The risk score for each sample was determined by the equation: Risk score = Σ (coefficient × expression value), where the coefficient corresponds to the regression coefficient and the expression value refers to the gene expression level. Additionally, the ‘ggplot2’ R package was utilized for data visualization, creating Sankey diagrams to illustrate the interrelations between the two risk clusters and the overall clustering patterns. For further validation, the dataset was segmented into overall, TCGA‐specific and GEO‐specific groups to conduct Kaplan–Meier survival analysis, generate ROC curves and carry out decision curve analysis (DCA).

### 2.5 Functional Enrichment Analysis and Immune Infiltration

Gene Ontology (GO) classification and Kyoto Encyclopedia of Genes and Genomes (KEGG) pathway enrichment analysis [15] were conducted to identify potential roles of differentially expressed genes (DEGs) in ARDS and non‐ARDS groups, with statistical significance set at FDR < 0.05. Utilizing R, the CIBERSORT and ESTIMATE algorithms were applied to discern variations in cohorts.

### 2.6 Copy Number Variation and Tumour Mutation Burden

Genomic Data Commons (GDC) Data Portal. Bar plots illustrating copy number variations and circos plots depicting chromosome positions were crafted using the ‘ggplot2’ R package. The ‘maftools’ R package was used to generate waterfall plots that delineate the mutational profiles of patients stratified into high‐risk and low‐risk groups. The association between mutational burden and risk scores was graphically represented using the ‘ggpubr’ and ‘reshape2’ R packages.

### 2.7 Single‐Cell Level Validation

The ‘Seurat’ package in R was utilized for the analysis of single‐cell RNA sequencing (scRNA‐seq) data. Initially, cells with ‘nFeature’ fewer than 200 and ‘percent.mt’ less than 20% were excluded as part of the data quality assessment. Subsequently, single‐cell data from different samples were integrated, and batch effects were mitigated. The ‘LogNormalization’ approach was used for the unsupervised clustering of cells before visualization utilizing principal component analysis (PCA) and t‐Distributed Stochastic Neighbour Embedding (t‐SNE). The ‘SingleR’ package facilitated the annotation of cell types in each cluster, whereas the ‘FindAllMarkers’ package was used to detect marker genes exhibiting varying expression levels across distinct cell types.

### 2.8 Animal Model

Six‐ to eight‐week‐old Sprague–Dawley rats were used. Group A served as the normal control group, receiving an equal amount of physiological saline solution by gavage. Paraquat (80 mg/kg) was diluted in 5 mL of physiological saline and administered by gavage as a one‐time dose for Group B, which served as the paraquat poisoning control group. After poisoning, Group B received an equal amount of physiological saline solution via tail vein injection for control. Group C was the glucocorticoid treatment group, receiving methylprednisolone (30 mg/kg) after poisoning. Group D was the glucocorticoid combined with the 20% fat emulsion treatment group, receiving 20% fat emulsion (5 mL/kg) and methylprednisolone via tail vein injection after poisoning. Groups C and D started daily drug intervention 6 h after PQ gavage, whereas Groups A and B received an equal amount of physiological saline solution tail vein injection for control at the same time point. Medication was administered once daily for all groups.

### 2.9 Measurement of Plasma Cytokine Levels

The above‐mentioned rats were subjected to different treatments, and blood was collected from the tail vein 1 day after treatment. The blood was collected in heparinized EP tubes. Radioimmunoassay technology was used to measure the levels of TNF‐α (Cat No. 88‐7340‐22, Invitrogen), IL‐1β (Cat No. PI303, Beyotime) and IL‐6 (Cat No. ERA31RB, Invitrogen) in the plasma and the concentrations of TGF‐β1 (Cat No. PT878, Beyotime) and PDGFB (Cat No. PP775, Beyotime) in the serum, following the instructions provided with the assay kits strictly [16, 17].

### 2.10 Double‐Immunofluorescence

First, the rats were given an intraperitoneal injection of 1% pentobarbital sodium anaesthesia, followed by cardiac perfusion. The rats were rinsed with normal saline and then rinsed with 4% formaldehyde for 10 min until limbs twitch and spine stiffens. Lung tissue was retrieved, fixed in 4% formaldehyde overnight at 4°C and embedded in OCT and the lung was sectioned in a coronal plane with a thickness of 20 μm. Blocking was performed with bovine serum and membrane permeabilization, incubated with primary antibodies (rabbit anti‐AE1, Cat No: #20112, CST, 1:100; rabbit anti‐PDGFB, Cat No: ab23914, abcam, 1:100 and rabbit anti‐IL‐1β, Cat No: ab283818, abcam, 1:100) overnight at 4°C and then washed with PBS three times. The samples were incubated with respective fluorescent secondary antibodies at 20°C for 2 h and washed with PBS to remove the secondary antibodies and the slides were prepared. All the images were captured using the Recordbio NE610 microscope.

### 2.11 Statistics

All statistical analyses were conducted using the R programming language (Version 4.0.3). Unless specified otherwise, a difference with a *p*‐value of less than 0.05 was deemed statistically significant.

## 3 Result

### 3.1 ARDS Related to the Prognosis of Pulmonary Adenocarcinoma

**FIGURE 1:**
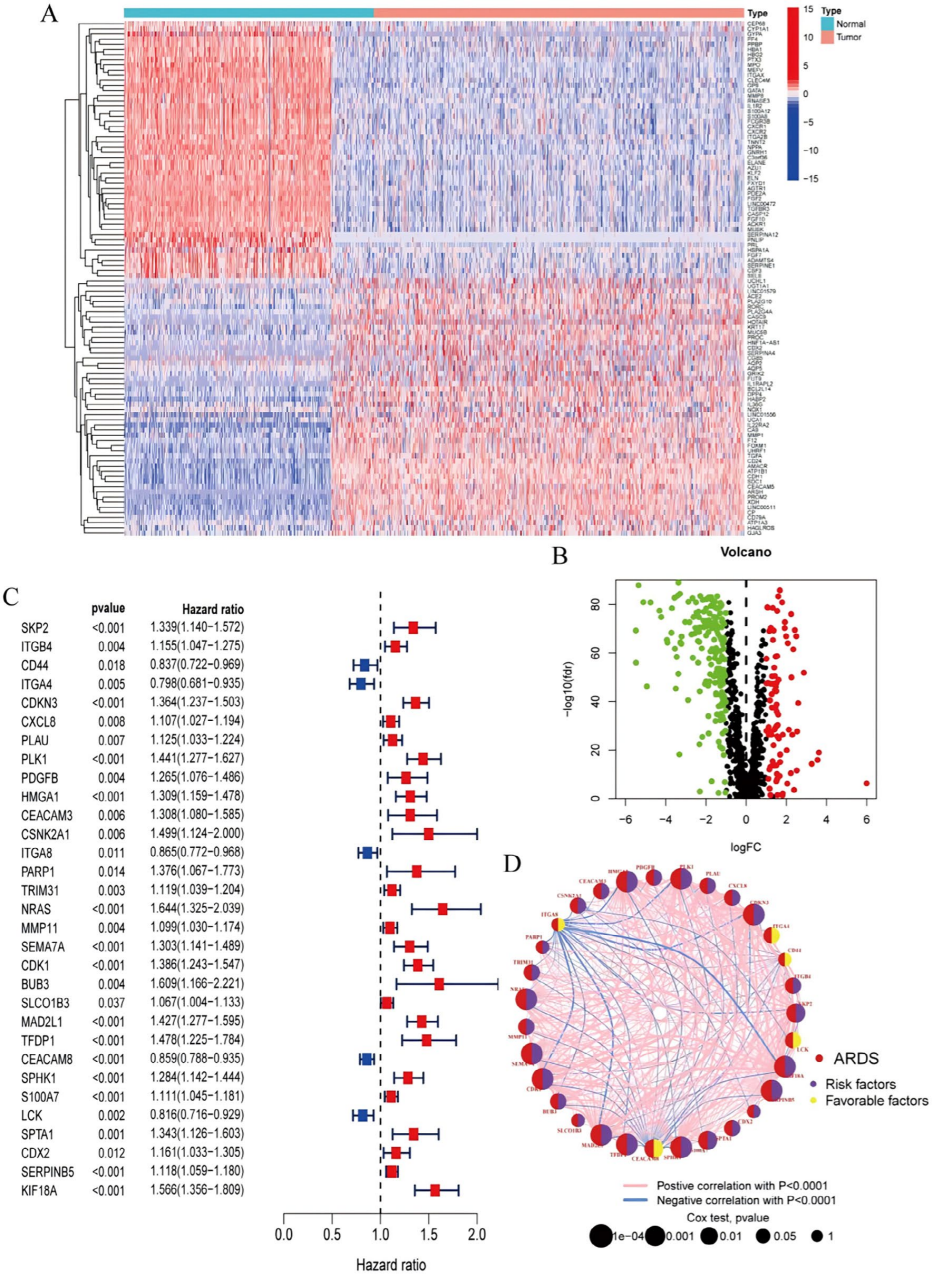
ARDS associated with prognosis of lung adenocarcinoma. (A) Heat map of differential genes. (B) Volcano plot of differential genes. (C) Forest plot of prognosis‐related genes. (D) Prognosis‐related gene correlation network.

A total of 276 ARDS‐related differential genes in LUAD were obtained through differential analysis of TCGA‐LUAD combined with GTEx and GEO (logFCfilter = 1, fdrFilter = 0.05), and heat maps and volcano plots were drawn (Figure 1A,B). Combining survival status and survival time, a single‐factor COX analysis of ARDS‐related differential genes in LUAD was conducted, obtaining a total of 31 prognosis‐related genes (Figure 1C). Subsequently, based on the expression matrix of prognosis genes, a correlation network between them was drawn (Figure 1D).

### 3.2 Consensus Clustering Analysis

**FIGURE 2:**
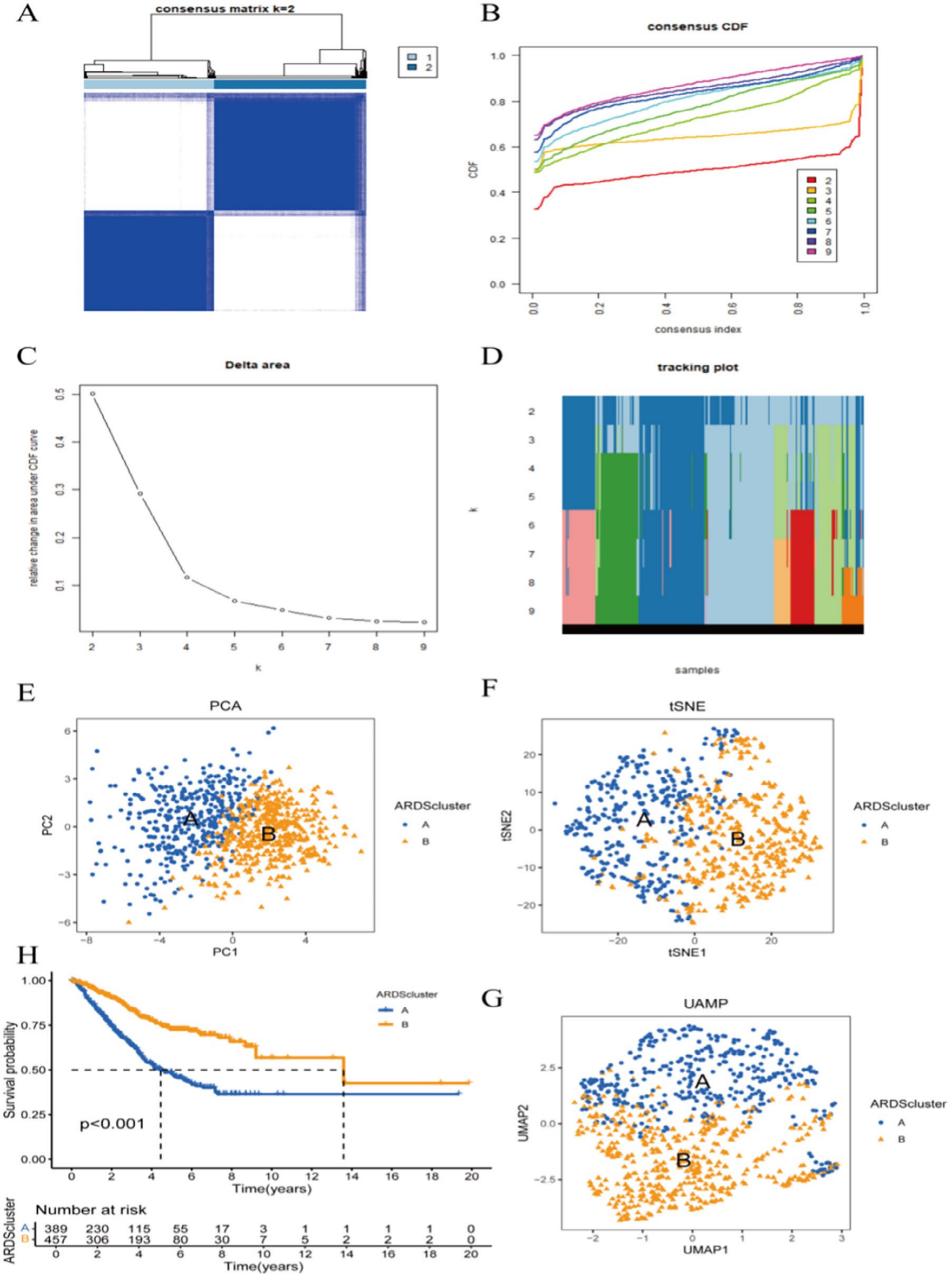
Consensus clustering analysis. (A) Consistency matrix heatmap. (B) Cumulative distribution function. (C) Delta area plot. (D) Tracking plot. (E) PCA plot. (F) t‐SNE plot. (G) UAMP plot. (H) Kaplan–Meier survival analysis.

Consensus clustering analysis was performed on samples from patients with LUAD to identify the ARDS‐sensitive subgroup. Through consensus clustering analysis, patients were divided into 2 subtypes. Based on the legend of the heat map generated by the consensus matrix, the optimal result was achieved when *K* = 2 (Figure 2A–D). Further principal component analysis and visualization of the clustering results showed that the ARDS group and the non‐ARDS group could be well distinguished (Figure 2E–G). Kaplan–Meier survival analysis demonstrated the differences in survival between the two groups, with poorer prognosis observed in the ARDS group (Figure 2H).

### 3.3 Machine Learning and Model Building

**FIGURE 3:**
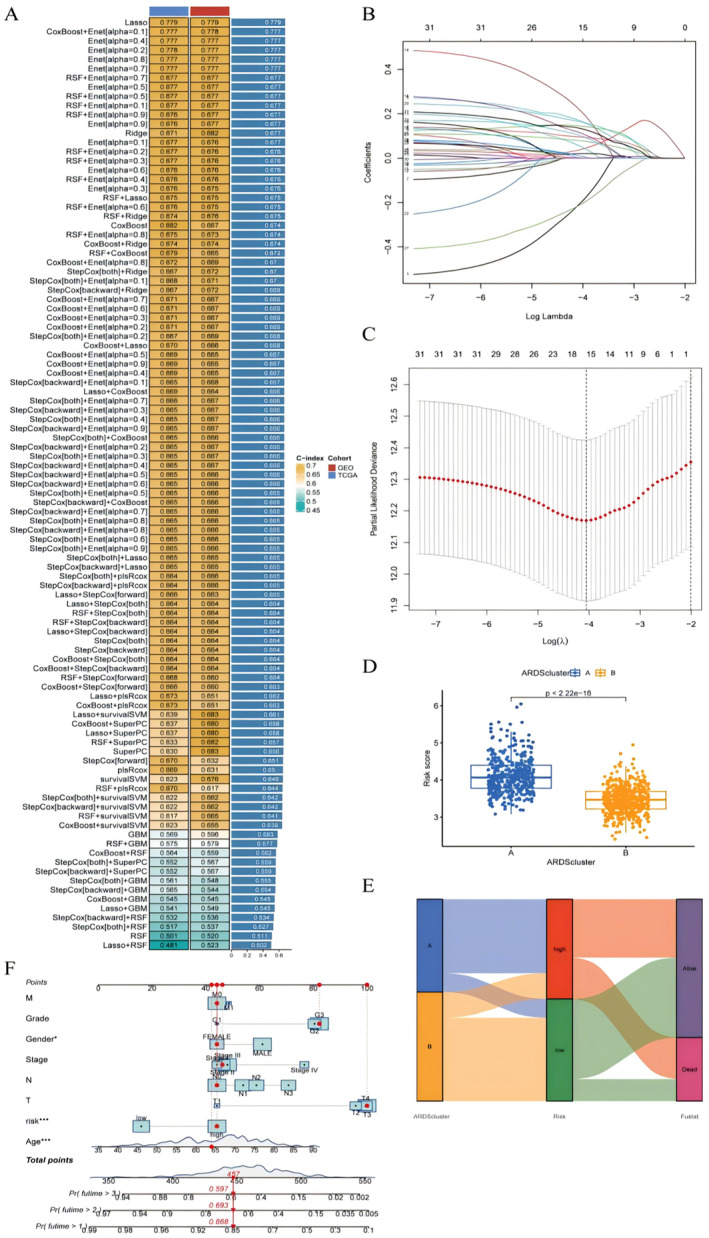
Machine learning and model building. (A) Heatmap of the C‐index of machine learning. (B) Lasso coefficient path plot. (C) Cross‐validation curve. (D) Box plot of risk score differences. (E) Sankey diagram mapping of risk scores and clustering. (F) Bar chart.*p < 0.05;**p < 0.01.

To build diagnostic models for ARDS and LUAD, we utilized a combination of 15 machine learning algorithms to analyse the prognostic‐related genes obtained above. The LUAD dataset was divided into TCGA and GEO based on the source. In TCGA, we fitted 101 predictive models using a tenfold cross‐validation framework and calculated the C‐index (Figure 3A). After comprehensive consideration, the Lasso model was selected, combined with the results of the lasso coefficient path plot and cross‐validation curve (Figure 3B,C), finally constructing a risk model composed of 15 genes Risk = (−0.20379322) + (0.09569914) + (0.16825783) + (0.03795813) + (0.14508527) + (0.05392615) + (0.21519908) + (0.11414774) + (0.07854581) + (0.10162127) + (0.02014629) + (−0.21066656) + (0.00969627) + (0.16422343) + (0.13414395). There were differences in risk scores between the ARDS group and the non‐ARDS group, with the ARDS group having higher risk scores (Figure 3D). Subsequently, we mapped the ARDS group and non‐ARDS group to the high‐risk and low‐risk groups and plotted a Sankey diagram. The results showed that the ARDS group and non‐ARDS group were consistent with the high‐risk and low‐risk groups (Figure 3E). Based on the clinical information, we created a bar chart and randomly selected a sample for display (Figure 3F).

### 3.4 Model Validation

**FIGURE 4:**
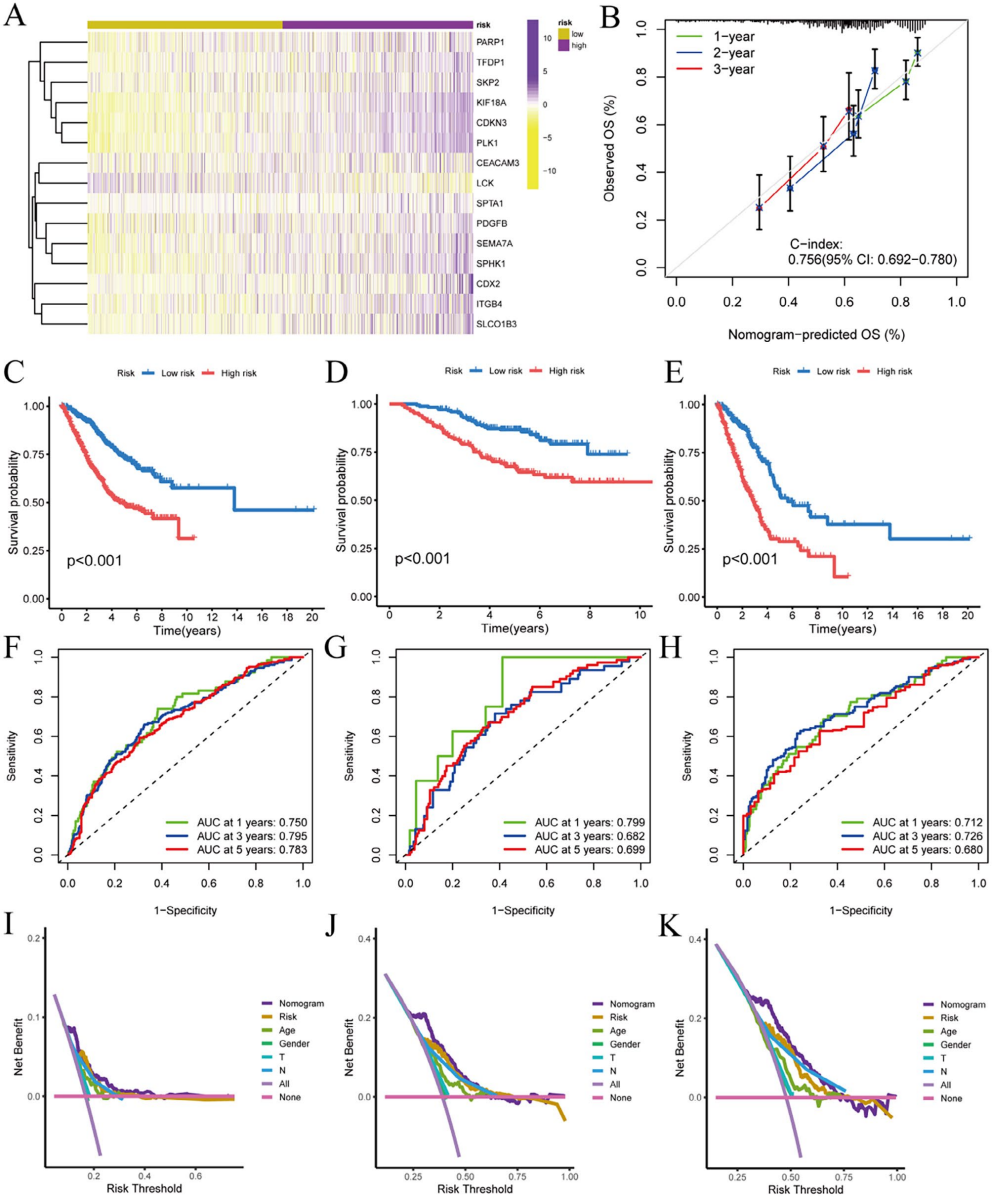
Model validation. (A) Differential heatmap of model. (B) Calibration plot for survival analysis. (C) Kaplan–Meier survival analysis (all samples). (D) Kaplan–Meier survival analysis (TCGA group). (E) Kaplan–Meier survival analysis (GEO group). (F) ROC analysis (all samples). (G) ROC analysis (TCGA group). (H) ROC analysis (GEO group). (I) DCA analysis (all samples). (J) DCA analysis (TCGA group). (K) DCA analysis (GEO group).

A differential heatmap of model genes was plotted between the two groups, showing that the expression trend is consistent with the model coefficients (Figure 4A). The C‐index for predicting LUAD patients' 1‐, 2‐ and 3‐year survival demonstrates good predictive accuracy (Figure 4B). TCGA group and GEO group for survival difference analysis, ROC analysis and DCA analysis. The results indicated that regardless of being in all groups, TCGA group or GEO group, the risk model could effectively assess the prognosis of LUAD (Figure 4C–K).

### 3.5 Functional Enrichment Analysis

**FIGURE 5:**
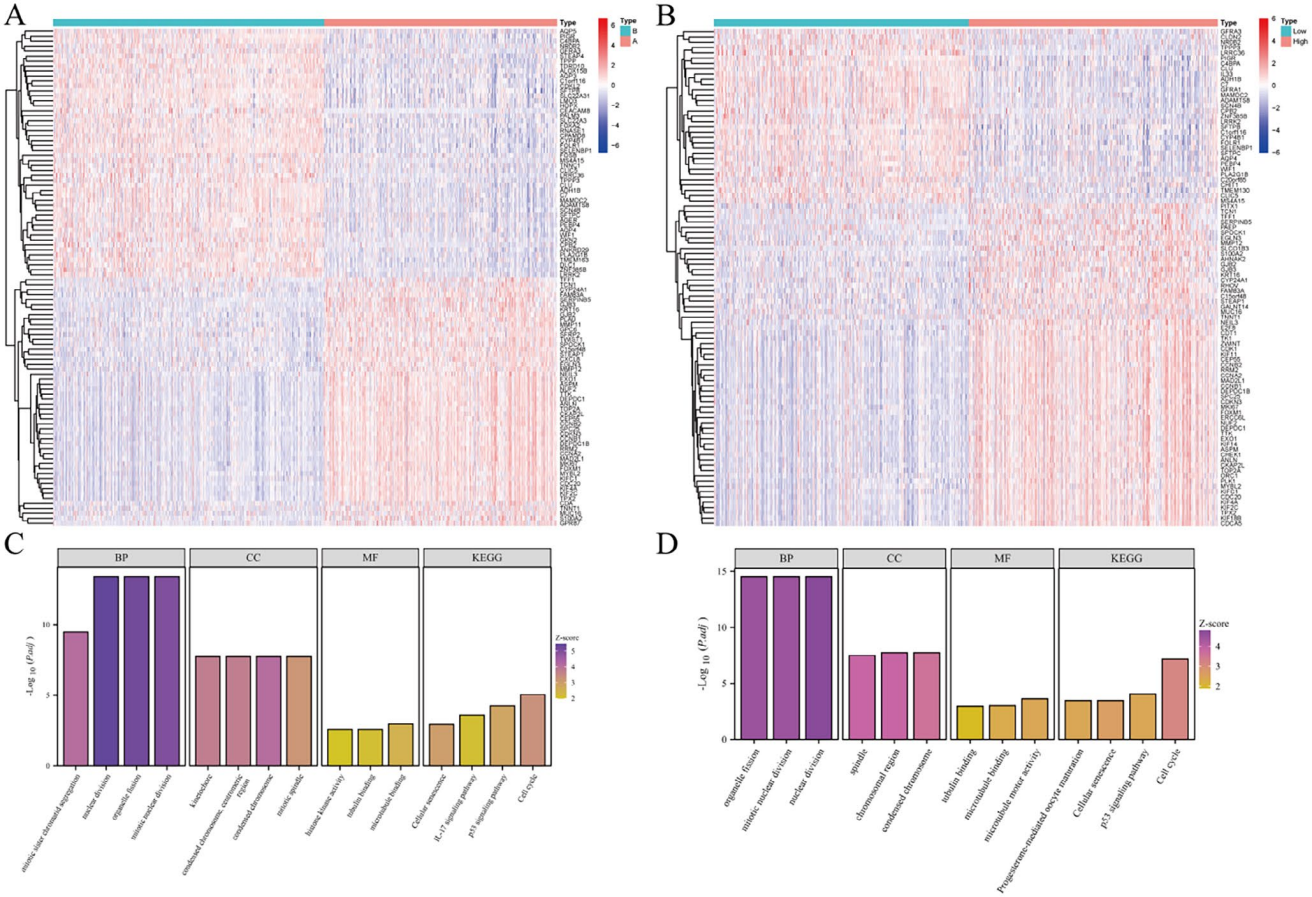
Functional enrichment analysis. (A, B) Heat map. (C, D) Bar graph of functional enrichment.

To further explain the potential mechanisms of consensus clustering analysis and risk models, differential analysis and KEGG and GO analyses were performed separately (Figure 5A,B). The results show that the potential mechanisms of the ARDS and non‐ARDS groups are highly similar to those. The enrichment results of cellular processes and cellular components. The KEGG results mainly focus on enrichment in the cell cycle and p53 signalling pathway (Figure 5C,D).

### 3.6 Immunological Analysis

**FIGURE 6:**
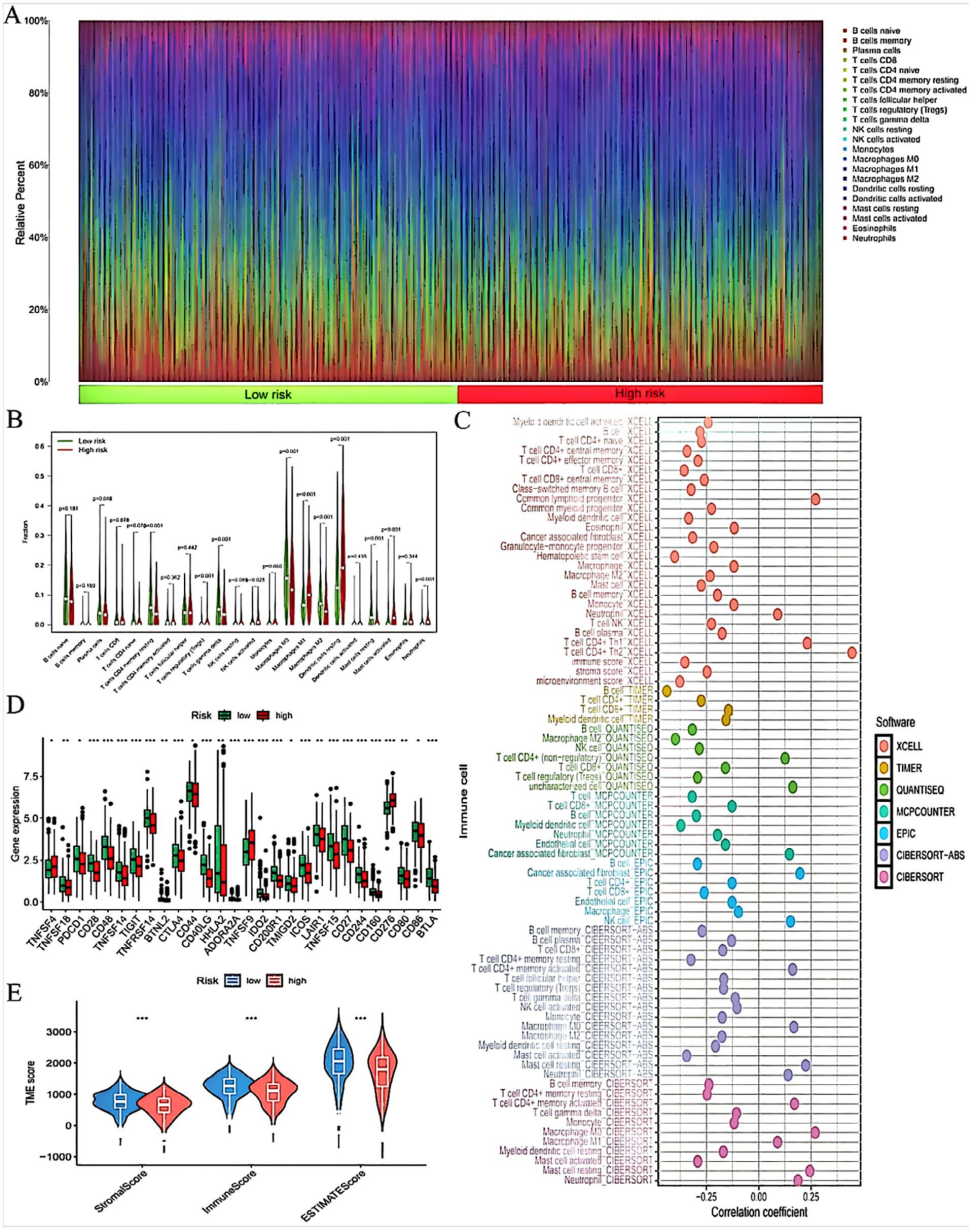
Immunological analysis. (A) Stacked bar graph of immune infiltration. (B) Violin plot of immune function. (C) Bubble plot of immune cell correlations. (D) Box plot of immune checkpoint expression. (E) Violin plot of stem cell score, immune score and microenvironment.

Immunological assessments were conducted for both high‐risk and low‐risk cohorts, utilizing the 22 immune cell markers available on the CIBERSORTx platform to determine the levels of immune cell infiltration within these groups (as depicted in Figure 6A). An integrated analysis of the functional disparities in immune cells (as shown in Figure 6B), along with an examination of the correlation between risk scores and immune cell profiles across various datasets (as illustrated in Figure 6C), indicated that the risk score predominantly affects natural killer cells and monocytes. A comparative analysis exposed significant variations in these checkpoints, which are intricately linked to the modulation of natural killer cells and monocytes in both risk groups (as detailed in Figure 6D). Moreover, a thorough scoring analysis of both groups revealed that the low‐risk group exhibited superior scores in stem cell, immune and microenvironment assessments, suggesting a higher likelihood of benefiting from immunotherapeutic interventions and a more favourable prognosis (as represented in Figure 6E).

While attempting to access the CIBERSORTx website for additional details, there may have been issues related to network connectivity or the link itself. If you intended to retrieve specific information from the website and encountered difficulties, the authors recommend verifying the URL's accuracy and attempting to access it again at a later time. If the website's content was crucial for your query and the issue persists, consider reaching out for technical support or exploring alternative resources that might offer similar immunological data.

### 3.7 Copy Number Variations and Mutation Burden

**FIGURE 7:**
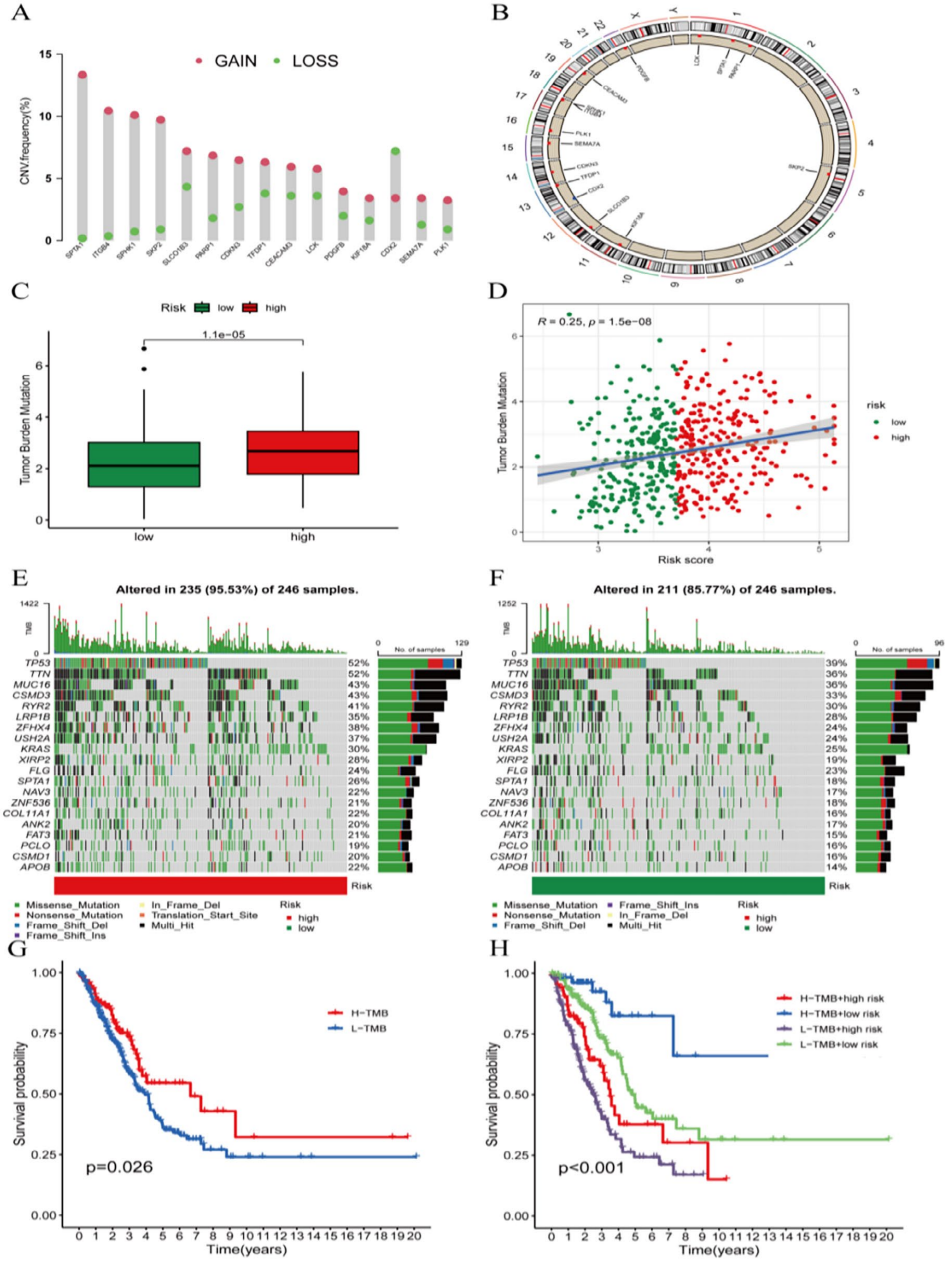
Copy number variations and mutation burden. (A) Bar graph of copy number variations of model genes. (B) Circular map of chromosome positions of model genes. (C) Box plot of mutation burden. (D) Scatter plot of risk score and mutation burden. (E, F) Waterfall plot of mutations. (G, H) Kaplan–Meier survival analysis.

Copy number variation and mutation burden information of LUAD samples were obtained from TCGA. Analysing the copy number variation trends of model genes in LUAD and drawing a chromosome position circular map (Figure 7B), the results show that except for CDX2 which shows copy number loss, the rest of the model genes exhibit copy number amplification (Figure 7A). Mutation burden analysis (Figure 7C) was performed, and the risk score is positively correlated with mutation burden (Figure 7D). The waterfall plots of mutation burden for high‐risk and low‐risk groups were created, showing that tumour‐related genes such as TP53, TTN and MUC16 have a higher mutation (Figure 7E,F). Survival analysis comparing high and low mutation burdens reveals better survival outcomes for the high mutation burden group (Figure 7G). Furthermore, combined survival prognosis analysis of high‐risk and low‐risk groups shows that the group (Figure 7H).

### 3.8 Preliminary Exploration of the Functional of Model Genes

**FIGURE 8:**
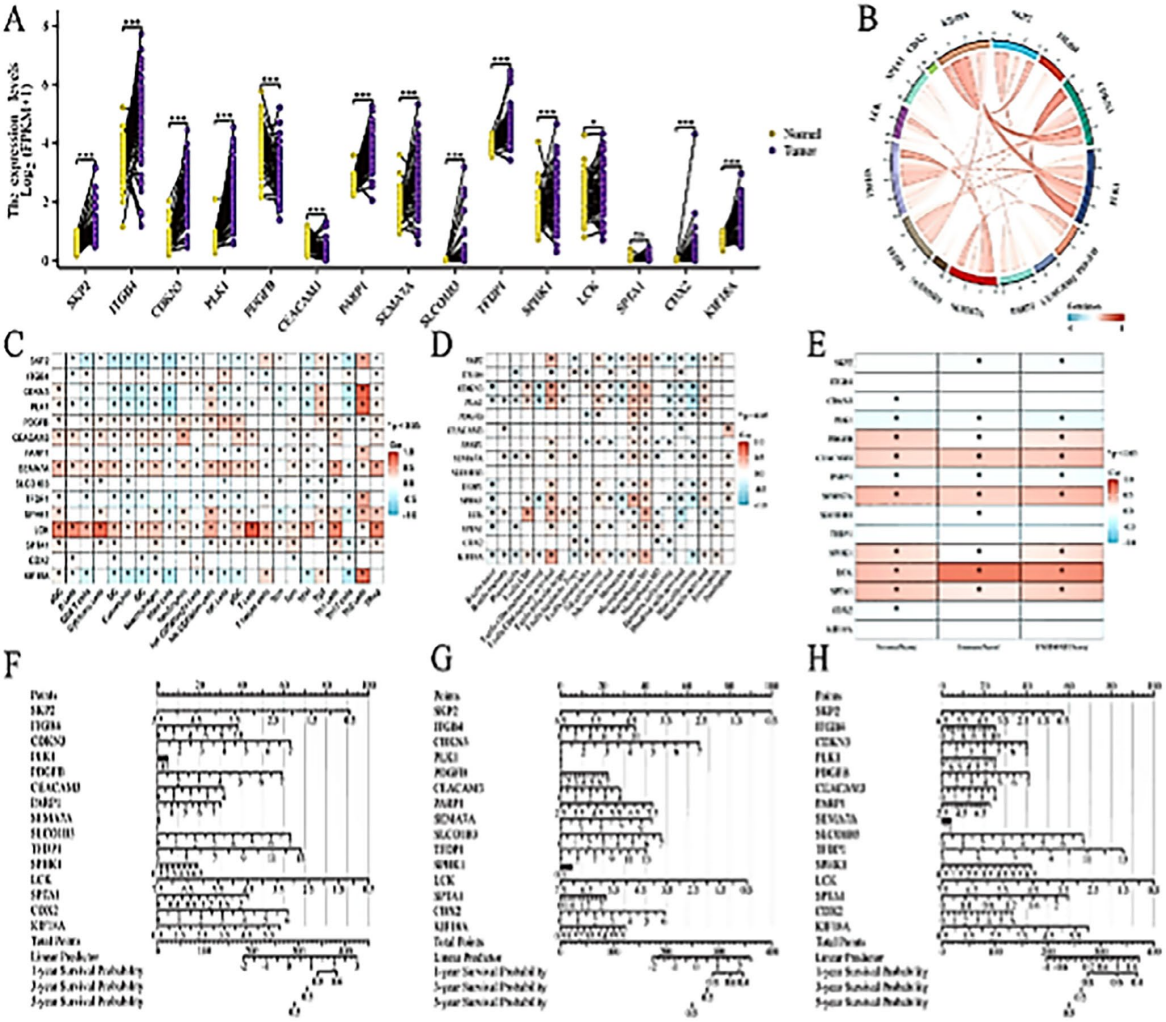
Preliminary exploration of the function of model genes. (A) Paired sample differential analysis of model genes. (B) Circular diagram of the correlation of model gene functions. (C) Heatmap of the correlation between model genes and immune cells. (D) Heatmap of the correlation between model genes and immune cell functions. (E) Heatmap of the correlation between model genes and stem cell score, immune score and tumour microenvironment. (F) Line chart (overall survival period). (G) Line chart (disease‐specific survival period). (H) Line chart (progression‐free interval).

Based on the clinical information from TCGA, paired sample differential analysis was performed on model genes, and the results showed that all genes except SPTA1 exhibited differences in paired samples, with trends consistent with the model coefficients (Figure 8A). Expression correlation analysis was conducted on model genes and a circular diagram was drawn, showing a positive correlation among the model genes (Figure 8B). Immunoinfiltration and tumour microenvironment correlation analysis were conducted on model genes, and a heatmap was drawn, indicating a close association between the model genes and numerous immune cells and their functions, mainly involving the expression and function of natural killer cells and monocyte‐derived macrophages (Figure 8C,D). Apart from SKP2, ITGB4, CDKN3, TFGP1, SLCO1B3, CDX2 and KIF18A, the remaining model genes showed significant correlations with stem cell score, immune score and tumour microenvironment (Figure 8E). Finally, line charts of 1‐, 3‐ and 5‐year survival in LUAD were plotted for model genes. The results indicated high scores for the model genes. Considering the paired sample differential analysis, expression correlation analysis, immunological and tumour microenvironment correlation analysis, as well as the line chart results, CEMACAM3, LCK, PARP1, PDGFB, PLK1, SEMA7A and SPHK1, were considered as prognostic genes of higher value.

### 3.9 Single‐Cell Level Validation

**FIGURE 9:**
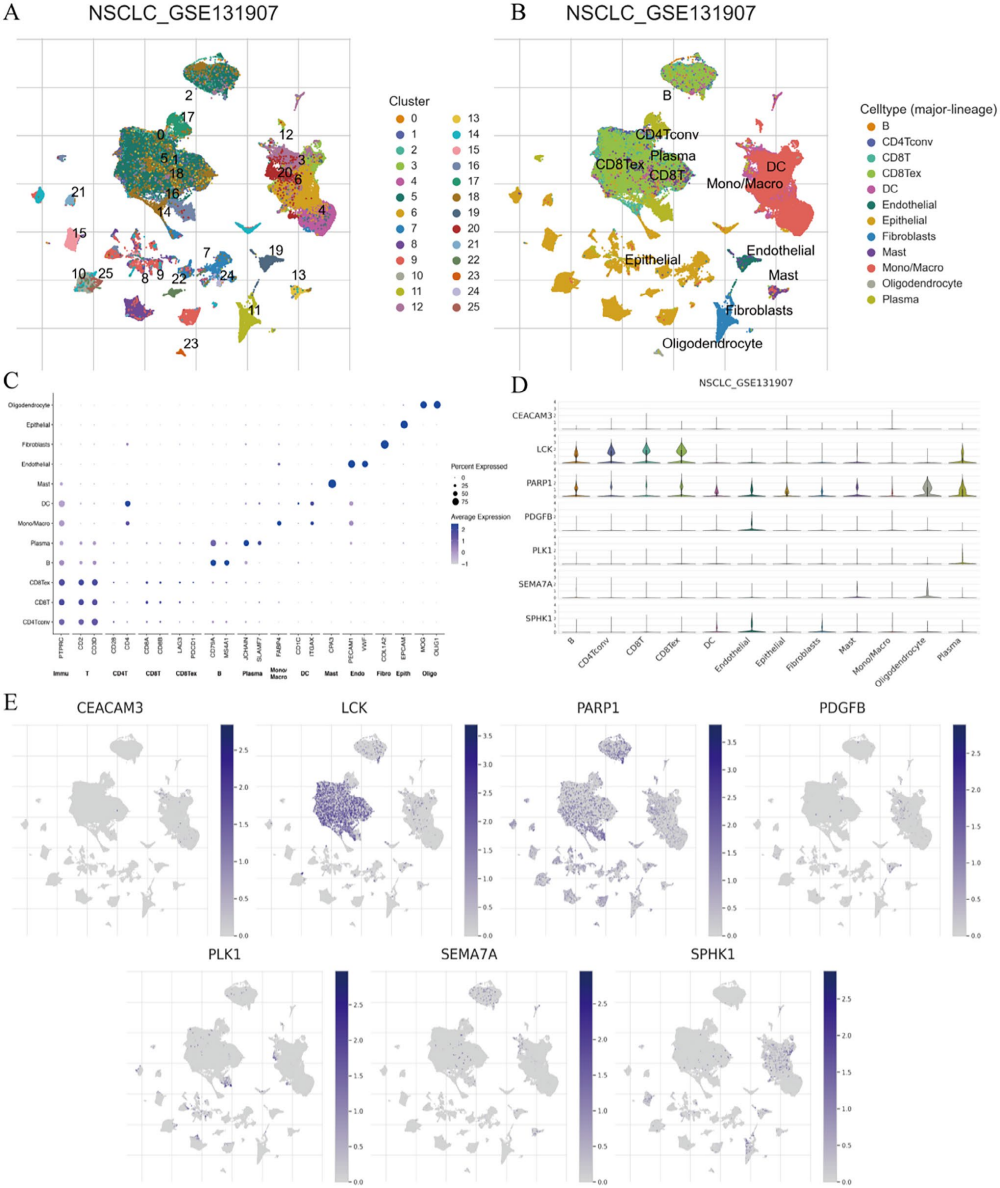
Single‐cell level validation. (A) After eliminating batch effects, organize t‐SNE clusters into cell clusters, with each hue representing a cell cluster. (B) After eliminating batch effects, visually sort t‐SNE clusters by known annotated cell types, with each colour representing a cell type. (C) Bubble plot of marker genes for each immune cell. (D) Expression of more valuable model genes in various immune cells (violin plot). (E) Expression of more valuable model genes in various immune cells (t‐SNE plot).

To further validate the results of the above verification and explore more detailed mechanisms, single‐cell level validation was conducted. Download GSE131907 from the GEO database, then merge, standardize and batch process the samples. t‐SNE clustering analysis was applied to the top 25 principal components, revealing that after accounting for batch effects, the visual representation of clustered results by sample origin indicates that the source of samples is no longer the predominant factor differentiating the cell groups (as shown in Figure 9A). SingleR was used to annotate cell clusters and interpret the t‐SNE clustering analysis (Figure 9B). Additionally, a bubble heatmap of SingleR annotated cell cluster marker genes with immune cells was plotted (Figure 9C). The analysis of more valuable model genes in various immune cells revealed that these genes are more expressed in CD8T, CD8Tex, CD4Tconv, macrophages, oligodendrocytes and plasma cells (Figure 9D). LCK and PARP1 are widely expressed in the above‐mentioned cells (Figure 9E).

### 3.10 Expression Levels of Inflammation‐Related Proteins in Plasma

**FIGURE 10:**
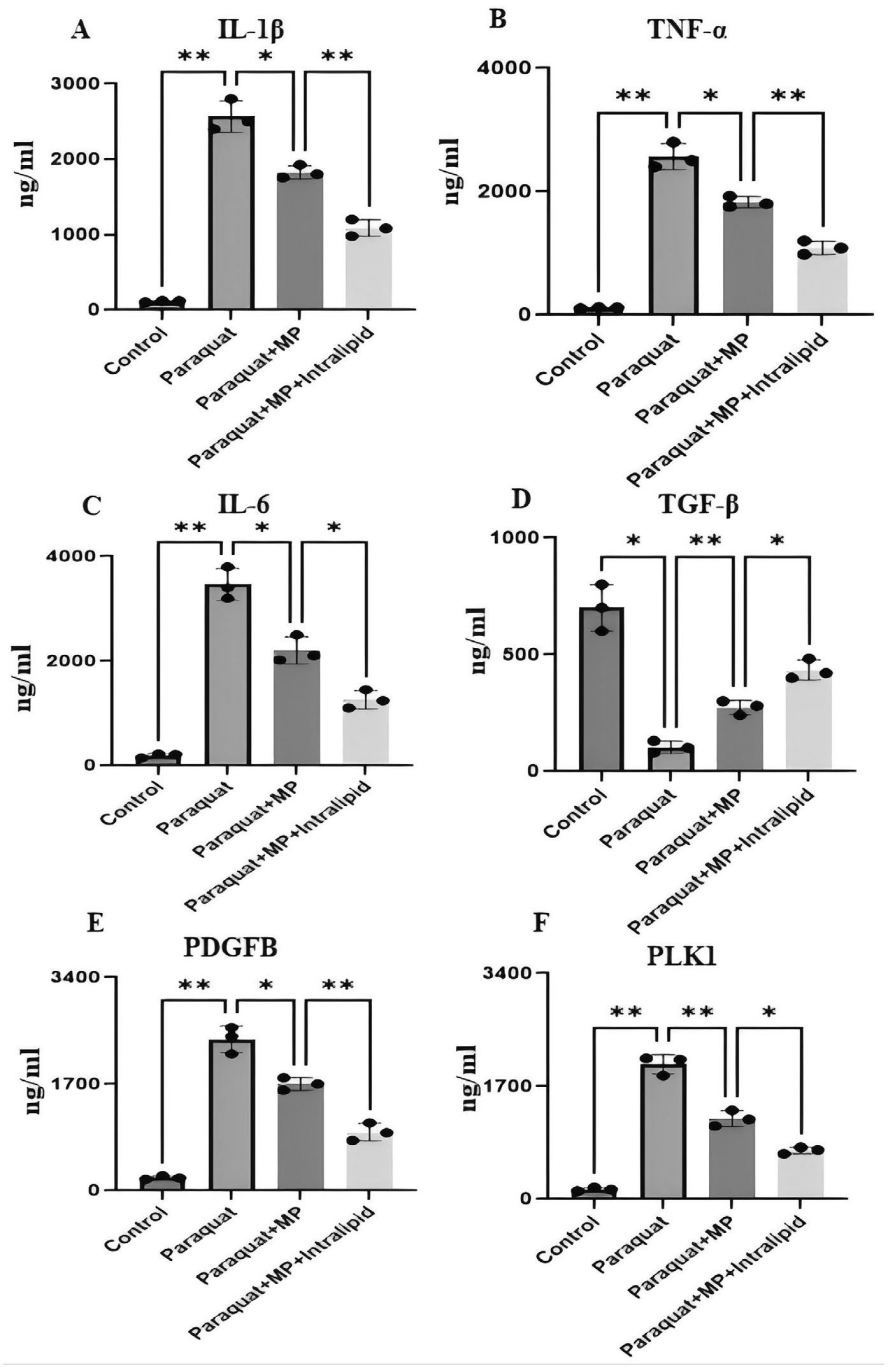
Expression levels of inflammation‐related proteins in plasma. (A–F) Histogram of expression levels of various inflammatory factors.

Compared with the control group, the group with paraquat poisoning showed increased expressions of IL‐1β, TNF‐α, IL‐6, PDGFB and PLK1, while after dexamethasone intervention, the expressions of IL‐1β, TNF‐α, IL‐6, PDGFB and PLK1 decreased. Intravenous fat emulsion further enhanced the effect of dexamethasone, and the expression trend of the anti‐inflammatory factor TGF‐β was opposite to that of IL‐1β (Figure 10A–F).

### 3.11 Expression of IL‐1β and PDGFB in Lung Tissue

**FIGURE 11:**
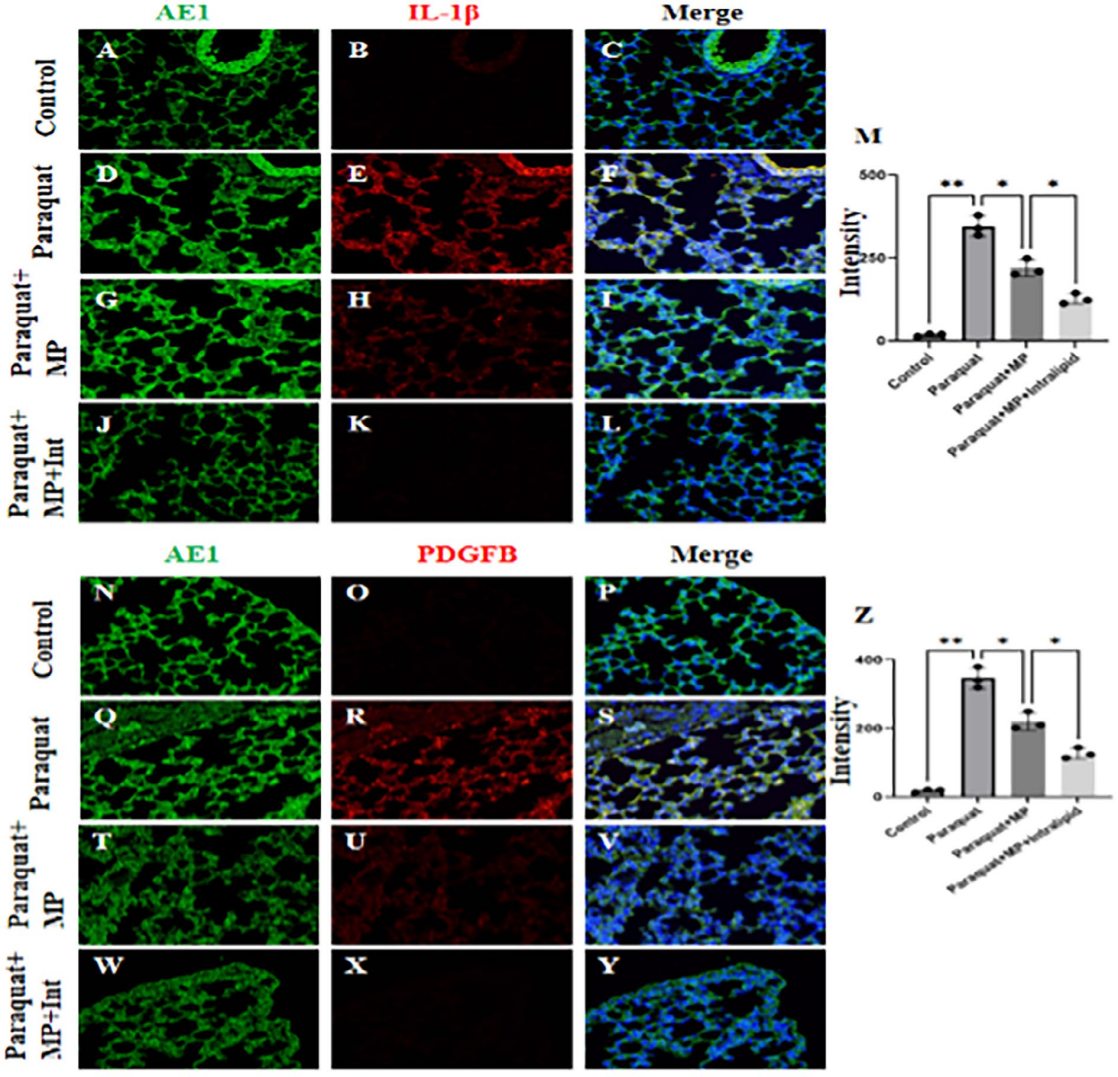
Expression of IL‐1β and PDGFB in lung tissue. The expression levels of IL‐1β and PDGFB in comparison to Day 1 levels within each group (A–G and N–Y). The paraquat‐poisoned group showed a notable rise in the average greyscale values of IL‐1β (D–F) and PDGFB (Q–S) in the lung tissues, which were effectively reversed following methylprednisolone treatment (G–I and T–V). The efficacy of methylprednisolone was further heightened with the addition of a fat emulsion (Figure J–M and W–Z).

In the lung tissues poisoned by paraquat, we compared the expression levels of IL‐1β and PDGFB with the expression levels at Day 1 in each group (Figures 11A–L, 11N–Y). In the paraquat‐poisoned group, the average greyscale values of IL‐1β (Figure 11D–F) and PDGFB (Figure 11Q–S) in the lung tissues were significantly increased, and this response was effectively reversed after treatment with methylprednisolone (Figures 11G–I, 11T–V). The action of methylprednisolone was further enhanced when an additional fat emulsion was added (Figures 11J–M, 11W–Z).

## 4 Discussion

This study comprehensively utilized data, as well as normal tissue data from GTEx. Through differential expression analysis, differentially expressed genes related to ARDS in LUAD were identified. The possible mechanisms through which ARDS affects LUAD involve inflammation and immune regulation. ARDS is a severe lung inflammation reaction, and the release of large amounts of inflammatory mediators in its pathophysiological process may promote the development and metastasis of LUAD [18, 19, 20]. In addition, the hypoxemia and alveolar‐capillary membrane damage caused by ARDS may provide a microenvironment favourable for the growth and invasion of tumour cells [21, 22]. During the treatment of LUAD, therapies such as chemotherapy or radiotherapy may induce ARDS, further impacting the prognosis of patients. These treatment modalities may indirectly promote the progression of tumours and resistance to treatment by affecting immune cells and cytokines in the lungs [23, 24, 25].

ARDS is a severe lung disease that can be caused by various factors, including infection, inhalation injury, trauma, inhalation of toxic gases and poisoning by certain drugs. Paraquat is a widely used herbicide with extremely high toxicity, even a small amount of ingestion can be fatal. Paraquat poisoning can lead to ARDS. After paraquat poisoning, the release of various cytokines (such as tumour necrosis factor α, interleukin‐1β, etc.) increases, and these cytokines are involved in the development of ARDS. Our research found that compared with the control group, the group with paraquat poisoning showed increased expressions of IL‐1β, TNF‐α, IL‐6, PDGFB and PLK1, while after dexamethasone intervention, the expressions of IL‐1β, TNF‐α, IL‐6, PDGFB and PLK1 decreased. IL‐1β and PDGFB in lung tissues were increased, and their expressions decreased after dexamethasone treatment. IL‐1β is a proinflammatory cytokine that plays a crucial role in the inflammatory response. In the context of ARDS, IL‐1β is released in large amounts in response to lung injury, contributing to the severe inflammation characteristic of the syndrome [26]. PDGFB can also promote the survival and proliferation of various cell types, including fibroblasts, which are key cells in the repair process. In the context of ARDS, this can lead to excessive fibrosis and a poor prognosis [27].

There are many potential pathways in which ARDS could impact the development of LUAD, including inflammation and immune regulation. ARDS lead to more vigorous inflammation and coronaviruses can produce significant numbers of proinflammatory mediators. In this inflammatory milieu, angiogenesis can be activated and there is increased support for the migration of epithelial cells undergoing EMT to become aggressive cancer subtypes able to evade immunity [28]. Furthermore, the hypoxemic state and the lung injury that accompanies ARDS can set the stage for tumour cell survival and metastasis. Hypoxia has been shown to cause genomic instability and adaptative selection, leading to aggressive phenotypes of tumour.

Functional enrichment analysis is mainly enriched in cell cycle control and cell division‐related pathways, which are closely associated with the tumour biological characteristics of LUAD [29, 30]. The abnormal expression of these genes may affect the proliferation and apoptosis of tumour cells, thereby influencing the progression and treatment response of the disease [31, 32]. The immune infiltration analysis indicates that these genes are closely related to the immune cell infiltration status in the tumour microenvironment, particularly natural killer cells and monocyte‐derived macrophages, providing potential targets for immunotherapy [30, 33, 34, 35]. The validation at the single‐cell level in the final section further corroborated the conclusions of immune infiltration and functional enrichment analysis. Copy number variation and mutation burden analysis further revealed the mutational characteristics of these genes in LUAD. We found that the model genes generally showed copy number increases, which may explain the phenomenon of high expression of oncogenes [36, 37]. Patients in the high‐risk group have a higher mutation burden, which may be related to the genetic instability of tumours, affecting the heterogeneity and treatment sensitivity of tumours [38, 39]. Furthermore, the positive correlation between the risk score and mutation burden suggests the interaction between gene expression and genetic variation in LUAD.

Genes correlated with stem cell scores may influence the plasticity and differentiation of cells within the lung tissue. In LUAD, alterations in these genes could lead to abnormal cell behaviour, potentially contributing to the inflammatory response and tissue damage that characterize ARDS. The immune score is related to the activation and infiltration of immune cells within the TME. Genes that correlate with immune scores may modulate the immune response to LUAD, which could either protect against or contribute to the development of ARDS, depending on the nature of the immune response.

The advantages of this study include a large sample size, integration of multiple data sources and multidimensional bioinformatics analysis, all of which enhance the reliability and comprehensiveness of the research findings. However, the study also has some limitations. First, it is primarily based on retrospective analysis, and further prospective studies are needed to validate these findings. Second, despite conducting various statistical and bioinformatics analyses, there may still be unidentified confounding factors that could affect the interpretation of the results. Additionally, this study mainly focuses on the genetic level of LUAD, and future research could explore changes in protein expression and metabolic pathways of ARDS‐related genes in LUAD.

The comprehensive analysis presented in this study not only enhances our understanding of the biological links between ARDS and LUAD but also offers a foundation for translating these insights into clinical practice. This could ultimately lead to improved diagnostics, more precise prognostication and the development of innovative treatment strategies for LUAD patients, particularly those at risk of ARDS.

Despite the use of advanced bioinformatics analyses, there may be unidentified confounding factors that could influence the results. These factors could include differences in patient demographics, comorbidities and variations in treatment protocols that were not fully accounted for in the analysis. The findings require validation in prospective studies to confirm their predictive value and to establish causal relationships between the identified genes and the development or progression of LUAD in the context of ARDS.

In conclusion, this study provides a new perspective on the role of ARDS‐related genes in the prognosis of LUAD and offers potential biomarkers for personalized treatment and prognosis assessment of LUAD. Future research will further explore the specific mechanisms of these genes in the development of LUAD and evaluate their application value in clinical practice.

## Author Contributions

**Erchun Hong:** data curation (equal), formal analysis (equal), writing – original draft (equal). **Yunyun Sun:** data curation (equal), formal analysis (equal), resources (equal), software (equal). **Yongming Qin:** data curation (equal), formal analysis (equal), software (equal). **Wenjun Zhao:** methodology (equal), validation (equal). **Yanzi Qin:** formal analysis (equal), visualization (equal). **Xincan Li:** investigation (equal), visualization (equal). **Liang Zhang:** funding acquisition (equal), project administration (equal), supervision (equal), visualization (equal), writing – review and editing (equal).

## Ethics Statement

This study was approved by the Experimental Animal Management and Ethics Committee of Bengbu Medical University, and all experimental animals and experimental operations in this study, were in accordance with the relevant national requirements for medical experimental animals, No.: Ethical Animal Section Approval No. [2023] 245.

## Conflicts of Interest

The authors declare no conflicts of interest.

## Data Availability

Data were obtained by contacting the corresponding author on request.
